# Population-based prevalence of malaria among pregnant women in Enugu State, Nigeria: the Healthy Beginning Initiative

**DOI:** 10.1186/s12936-015-0975-x

**Published:** 2015-11-05

**Authors:** Jayleen K. L. Gunn, John E. Ehiri, Elizabeth T. Jacobs, Kacey C. Ernst, Sydney Pettygrove, Lindsay N. Kohler, Steven D. Haenchen, Michael C. Obiefune, Chinenye O. Ezeanolue, Amaka G. Ogidi, Echezona E. Ezeanolue

**Affiliations:** Department of Epidemiology and Biostatistics, Mel and Enid Zuckerman College of Public Health, University of Arizona, 1295 N Martin Ave Drachman Hall, PO Box 245163, Tucson, AZ 85724 USA; Department of Health Promotion Sciences/Global Health Institute, Mel and Enid Zuckerman College of Public Health, University of Arizona, Tucson, AZ USA; University of Arizona Cancer Center, Tucson, AZ USA; Prevention, Education, Treatment, Training and Research-Global Solutions-PeTR-GS, Enugu, Enugu State Nigeria; Healthy Sunrise Foundation, Castle Ridge Avenue, Las Vegas, NV USA; Global Health and Implementation Science Initiative, School of Community Health Sciences, University of Nevada, Las Vegas, NV USA

**Keywords:** Prevalence, Pregnancy, Malaria, Nigeria

## Abstract

**Background:**

Malaria adversely affects pregnant women and their fetuses or neonates. Estimates of the malaria burden in pregnant women based on health facilities often do not present a true picture of the problem due to the low proportion of women delivering at these facilities in malaria-endemic regions.

**Methods:**

Data for this study were obtained from the Healthy Beginning Initiative using community-based sampling. Self-identified pregnant women between the ages of 17–45 years were recruited from churches in Enugu State, Nigeria. Malaria parasitaemia was classified as high and low based on the malaria plus system.

**Results:**

Of the 2069 pregnant women for whom malaria parasitaemia levels were recorded, over 99 % tested positive for malaria parasitaemia, 62 % showed low parasitaemia and 38 % high parasitaemia. After controlling for confounding variables, odds for high parasitaemia were lower among those who had more people in the household (for every one person increase in a household, OR = 0.94, 95 % CI 0.89–0.99).

**Conclusion:**

Results of this study are consistent with hospital-based estimates of malaria during pregnancy in southeastern Nigeria. Based on the high prevalence of malaria parasitaemia in this sample, education on best practices to prevent malaria during pregnancy, and resources in support of these practices are urgently needed.

## Background

Nigeria accounts for roughly 25 % of the malaria burden in sub-Saharan Africa [1, 2]. Often undetected and untreated, malaria adversely affects pregnant women and their fetuses or neonates [3–6]. Current estimates of malaria parasitaemia in Nigerian pregnant women vary greatly among geographic regions. Hospital-based prevalence percentages range from 5 % in the northwestern region [7], 17 % in the southwestern region [8], to 95 % in the southeastern region where Nigeria borders the Gulf of Guinea [9].

In Nigeria, only an estimated 35 % of pregnant women deliver at a healthcare facility [10]. Therefore, health facility-based estimates of the malaria burden in pregnant women often do not present a true picture of the problem. This cross-sectional study of pregnant women in the community is likely to be a more representative sample and, therefore, a more accurate estimate of the burden of malaria during pregnancy in southeastern Nigeria. The aims of this study were twofold: (1) to investigate the population-based malaria parasitaemia burden during pregnancy in Enugu State, Nigeria; and, (2) to explore person-level maternal risk factors that are associated with high malaria parasitaemia.

## Methods

Data for this study were obtained at baseline from the Healthy Beginning Initiative (HBI), which has previously been described in detail [11]. Briefly, the parent study was a two-arm cluster randomized trial in Enugu State, Nigeria aimed to assess rates of HIV testing. The HBI utilized a congregational-based recruiting method that first occurred at the level of the church, then the participant level. Bishops overseeing the dioceses were first approach for permission to talk to priests corresponding to the Bishop’s diocese. All priests allowed for recruitment within their corresponding congregations (n = 40). Self-identified pregnant women between the ages of 17 and 45 years were recruited beginning Jan 20, 2013, and completed by Sept 29, 2013 from 40 churches of varying denominations in Enugu State, Nigeria where the population is more than 95 % Christian. Recruitment from churches was expected to provide a representative sample of pregnant woman in Enugu State, Nigeria, as church attendance approaches 90 % in the country [11, 12]. All women who self-identified as pregnant were allowed into the study, no follow-up pregnancy test was required for pregnancy verification. Each Sunday, priests asked pregnant women and their male partners to come to the altar so the priest could perform a prayer that included encouraging women to seek care at a healthcare facility, for the woman to have healthy pregnancy, and for successful delivery of her fetus. He then introduced the HBI study as a resource that could be utilized by pregnant women in the congregation. Pregnant women were given information on the study and, if interested, were asked to read and sign a written consent form. The majority of participants were unable to read the local language (Ibo) and preferred to have study material in English. For participants who could not read, a church-based health advisor or a research assistant read the consent form aloud in English or Ibo; the participants gave their consent by affixing thumbprints or using initials. Because pregnant women were not directly approached for participation in the study, the number of pregnant women in the corresponding churches who did not wish to participate in the study is unknown.

A structured questionnaire consisting of both validated and not validated measures was implemented at a Sixth Grade reading level [11]. Trained research staff and church-based health advisors administered the survey. Participants had the option of reading the survey themselves or having study personnel read the questions to them in either English or Ibo. Other survey questions and laboratory measures not discussed in this paper can be found in the study protocol [11]. Only data ascertained during the baseline survey were utilized for this analysis.

Parasitaemia levels were assessed using the malaria plus system [13]. Thick blood smears were examined using microscopy under oil immersion [13]. To ensure quality control, each slide was examined by multiple laboratory technicians and random checks were made by a hospital review panel. The malaria plus system was scored as follows: 0 for no parasites, + for 1–10 parasites per 100 high power field, ++ for 11–100 parasites per 100 high power field, +++ for 1–10 parasites per high power field, ++++ for over 10 parasites per high power field. Thus, as levels of parasitaemia increase, the scoring moves from 0 through ++++ .

Malaria parasitaemia was classified as high and low based on the malaria plus system. Those in the 0 and + group were classified as low parasitaemia; while those in the ++, +++, and ++++ groups were classified as high parasitaemia. Gravidity was dichotomized as >3 previous pregnancies and <3 pregnancies. Associations between malaria parasitaemia and continuous variables were determined using ANOVA. Pearson’s Chi square test was used to examine associations of malaria with categorical and dichotomous variables. Crude and adjusted logistic regression models were used to determine the association between participant characteristics and malaria parasitaemia levels. All variables were retained in the final model. Statistical significance was set at p < 0.05. Data analyses were conducted using Stata version 12.0 (Stata Corporation, College Station, TX, USA). The parent study was approved by the Institutional Review Board of the University of Nevada, Reno, and the Nigerian National Health Research Ethics Committee. This secondary data-analysis was considered exempt from human subjects review by the Mel and Enid Zuckerman College of Public Health Research Office.

## Results

Malaria parasitaemia levels were recorded for 2069 pregnant women. Over 99 % of the women in the study tested positive for malaria parasitaemia (n = 2052). Categorized according to the malaria plus system, malaria parasitaemia in this sample included: fewer than <1 % no infection (n = 17); 62 % in the + group (n = 1275); 36 % in the ++ group (N = 737); 2 % in the +++group (n = 40); with no one falling into the ++++ group (n = 0) (see Table 1). When malaria was categorized as low and high parasitaemia, 62 % (n = 1292) were classified as low parasitaemia and 38 % (n = 777) as high parasitaemia. As shown in Table 2, no significant differences were found between high and low malaria parasitaemia and gravidity, area of residence, distance to nearest healthcare facility, antenatal care, household size, or age of the participants. Table 3 shows the results of logistic regression analysis of the association between participant characteristics and malaria parasitaemia. After controlling for confounding variables, odds for high parasitaemia were lower among those who had more people in the household (for every one person increase in a household, OR = 0.94, 95 % CI 0.89–0.99).

**Table 1:** Malaria parasitaemia frequency by the malaria plus system

Malaria plus system	N (%)
0	17 (<1)
+	1275 (62)
++	737 (36)
+++	40 (2)
++++	0 (0)
Total	2069 (100)

Levels of parasitaemia increase as the scoring moves from 0 through ++++

**Table 2:** Comparison of participants′ characteristics by malaria parasitaemia low vs. high

	Parasitaemia			
	Low	High	Total	p*
Number of participants, N (%)	1292 (62)	777 (38)	2069 (100)	
Gravidity, N (%)^a^				
Primi/secundigravida	819 (65.8)	486 (65.9)	1305 (65.5)	0.67
Multigravida	425 (34.2)	263 (35.1)	688 (34.5)	
Residence, N (%)^b^				
Urban	326 (25.3)	189 (24.1)	513 (24.9)	0.54
Rural	960 (74.7)	588 (75.9)	1548 (75.1)	
Distance to healthcare facility, N (%)^c^				
0–5 (km)	464 (36.1)	255 (33.0)	719 (34.9)	0.17
5–10 (km)	487 (37.9)	290 (37.5)	777 (37.8)	
10+ (km)	334 (26.0)	228 (29.5)	576 (27.3)	
Antenatal care, N (%)^d^				
Yes	1014 (78.5)	627 (80.7)	1641 (79.3)	0.23
No	278 (21.5)	150 (19.3)	428 (20.7)	
Household size, mean (SD)^e^	4.51 (2.0)	4.36 (1.9)	4.40 (1.9)	0.07
Age (Years), mean (SD)^f^	29.10 (5.8)	28.90 (5.8)	29.00 (5.7)	0.44

* Significance based on Chi square and ANOVA *p* value <0.05

^a^Missing 48 low parasitaemia, 28 high parasitaemia

^b^Missing 6 low parasitaemia

^c^Missing 7 low parasitaemia, 4 high parasitaemia

^d^No missing data

^e^Missing 10 low parasitaemia, 9 high parasitaemia

^f^No missing data

**Table 3:** Logistic regression models for malaria parasitaemia and participant level characteristics

	N	Crude model	Adjusted^a^
		OR (95 % CI)	OR (95 % CI)
Gravidity			
Primi/secundigravida	688	1.04 (0.86–1.26)	0.93 (0.75–1.17)
Multigravida	1305	Ref.	Ref.
Residence			
Urban	513	Ref.	Ref.
Rural	1548	1.07 (0.87–1.31)	1.07 (0.86–1.32)
Distance to healthcare facility			
0–5 (km)	719	Ref.	Ref.
5–10 (km)	777	1.08 (0.88–1.34)	1.09 (0.87–1.35)
10+ (km)	562	1.24 (0.99–1.56)	1.23 (0.97–1.55)
Antenatal care			
Yes	1641	Ref.	Ref.
No	428	0.87 (0.70–1.09)	0.84 (0.66–1.05)
Household size	2050	0.96 (0.91–1.00)	0.94 (0.89–0.99)^b^
Age	2069	0.99 (0.98–1.01)	1.00 (0.98–1.02)

^a^Overall model *n* = 1970

^b^Indicates significance at p < 0.05

## Conclusion

The results of this study demonstrated that over 99 % of pregnant women in the HBI showed some level of malaria parasitaemia, with 38 % showing high levels of parasitaemia. For each additional person in the household, a 6 % lower odds of high malaria parasitaemia was found.

Estimates presented in this paper are consistent with hospital-based estimates of malaria during pregnancy in the southeastern region of Nigeria [9]. Malaria places a heavy burden on Nigeria’s fragile healthcare system with nearly 110 million clinical cases occurring a year, accounting for up to 60 % of outpatient visits and 30 % of hospital admissions [1]. The Roll Back Malaria programme supports the WHO recommendations that pregnant women receive intermittent preventative treatment with the inclusion of sulfadoxine-pyrimethamine (IPTp) as part of antenatal care and recommends that pregnant women sleep under insecticide-treated nets (ITN). Although Nigeria has adopted these recommendations, it has a long way to go in achieving these goals with only 13.2 % of pregnant women receiving IPTp and 33.7 % of pregnant women sleeping under an ITN [1, 14]. Although approximately 80 % of women in this study reported receiving antenatal care, the quality of this care remains unknown. However, because this study demonstrated a high prevalence of malaria parasitaemia, IPTp and ITN are thought to either not be an integral part of antenatal care or barriers remain for women who participated in this study to receive or use IPTp and/or ITN. Future studies are warranted that assess the reasons for the high prevalence of malaria parasitaemia during pregnancy in this region.

Results from this study indicated that household size significantly influenced malaria parasitaemia with lower odds of high parasitaemia with each additional person in the household. Because immunity to malaria has been demonstrated to increase with each additional pregnancy, it is likely that the number of people in the household may be an indirect measure of her parity [15, 16]. Therefore, the decrease in the odds of high malaria parasitaemia with an increase in the number of people in the household demonstrated in this study maybe indicative of past immune response to malaria during pregnancy. One other explanation of this association between household size and increased parasitaemia may be found from Watsierah and colleagues who demonstrated that households with three or more people had a higher proportion of individuals in the household taking anti-malarial drugs at the correct dosage and duration than smaller households [17]. Information on anti-malarial medication was not ascertained in the present study, however; therefore, it is unknown if this is a likely explanation for these results.

This study is not without limitations. The proportion of pregnant women who demonstrated clinical malaria symptoms was not recorded. Also, the proportion of women who were sleeping under an ITN or taking IPTp is unknown. Because over 99 % of women in this study had malaria parasitaemia, it may be assumed that few women were taking IPTp or were sleeping under an ITN; however, this cannot be confirmed.

Based on the high prevalence of malaria in this sample, education on best practices to prevent malaria during pregnancy, and resources in support of these practices are urgently needed. Organizations aimed at reducing malaria in southeastern Nigeria may want to consider using congregations to distribute anti-malarial resources as this study demonstrated high rates of malaria parasitaemia in this population.
